# A Rare Case of Bilateral Foot Drop Following Cervical Decompression in Tandem Spinal Stenosis

**DOI:** 10.7759/cureus.76581

**Published:** 2024-12-29

**Authors:** Muhammad Ishfaq, Rajeesh George, Gamaliel Tan

**Affiliations:** 1 Orthopaedic Surgery, Ng Teng Fong General Hospital, Singapore, SGP

**Keywords:** cervical decompression, cervical spondylotic myelopathy, foot drop, ischemia reperfusion injury, tandem stenosis

## Abstract

This case report describes a 70-year-old male presenting with limb weakness, urinary retention and tandem cervical and lumbar spinal stenosis with complicating white cord syndrome, a rare reperfusion injury post decompression surgery. Initially admitted following an unwitnessed fall, the patient's neurological examination indicated that progressive weakness of the limbs and sensory loss etiology is cervical and lumbar spondylosis with severe spinal canal stenosis, confirmed by imaging. Due to rapid deterioration, he underwent C5 corpectomy, cervical decompression and fusion. Informed consent for surgery was obtained from the patient. Post-surgery, he experienced transient improvements but soon developed delirium, worsening right-sided weakness, and bilateral foot drop. Diagnosis of white cord syndrome was made because of repeat cervical MRI findings having signal changes in cervical spine.

Subsequent treatment included intravenous steroids, antibiotics, and eventual lumbar interbody fusion. The multifactorial nature of his postoperative complications including hyperactive delirium and urinary tract infection, underscores the complexities associated with tandem stenosis and white cord syndrome management. The case highlights the need for early intervention in tandem spinal stenosis cases, cautious intraoperative monitoring, and risk factors management for reperfusion injury, stressing the role of comprehensive postoperative care to improve functional outcomes.

## Introduction

Tandem spinal stenosis is a rare clinical condition, with prevalence in the literature reported between 5% and 28% and there is variability in its prevalence because different studies were conducted in different setups with different patients' demographics. It results from degenerative changes that lead to significant narrowing of the spinal canal at multiple levels, causing symptomatic compression of the spinal cord. Typically, when surgery is performed on the most symptomatic region, signs and symptoms in less symptomatic areas may subsequently emerge while the hallmark symptoms of cervical spondylotic myelopathy include gait instability, loss of hand dexterity, and sensory and motor dysfunction in both the upper and lower limbs [1-4].

Cervical spinal cord compression and resulting neuronal damage arise through mechanisms broadly categorized as dynamic and static [5,6]. Static risk factors, such as intervertebral disc prolapse, osteophyte formation, hypertrophy of the ligamentum flavum, and facet joint enlargement, contribute to progressive spinal cord injury through central canal tightening, resulting in local ischemia, neuronal injury, and apoptosis. Dynamic factors involve repetitive injuries to neuronal tissues from cervical spine flexion and extension movements, which stretch the axons and increase their vulnerability to further damage [7-9].

MRI provides detailed sagittal and axial views of spinal stenosis severity, with high signal detection on MRI suggesting permanent spinal damage or myelomalacia [10]. Computed tomography (CT) offers additional information on bone structures, osteophytes, and ossified posterior longitudinal ligament that may contribute to spinal canal stenosis. Myelography can be used for surgical planning if MRI results are inconclusive or contraindicated [11]. For pronounced myelopathy seen clinically and radiologically, decompression via anterior or posterior approaches is strongly advised to halt disease progression [12,13].

Lumbar spinal stenosis is primarily due to age-related degenerative changes in the facet joints, ligamentum flavum, and intervertebral discs, leading to spinal canal narrowing and subsequent compression of neurovascular structures. This results in chronic pain and disability, severely impacting the patient’s quality of life, mobility, and functionality [14,15]. Although X-ray and CT can demonstrate degenerative changes, MRI remains the most accurate diagnostic tool to assess central spinal canal and foraminal stenosis severity [16]. Surgery aims to decompress neurovascular structures and, if needed, stabilize the spine through fusion [17].

Tandem spinal stenosis, involving compression at multiple spinal levels, can lead to neurological deficits. Clinicians should remain vigilant for new symptom development following successful surgical intervention for tandem spinal stenosis [18].

A rare complication of cervical spine decompression surgery is spinal cord reperfusion injury, also known as "white cord syndrome" (WCS). Although the mechanism of the disease is unclear, it is postulated that ischemic reperfusion injury occurs when blood flow is restored to previously ischemic tissues and organs, causing the formation of oxygen-free radicals that damage the spinal cord and a disruption of the blood-spinal cord barrier [19].

## Case presentation

A 70-year-old Chinese male with a medical history of hypertension, diabetes mellitus, bilateral hearing loss, and bilateral total knee replacements was admitted to the general medicine department via the emergency room following an unwitnessed fall. He reported progressive bilateral weakness in both his upper and lower limbs over the past three months. On neurological examination, he exhibited 5/5 power in all key myotomes of the four limbs, with reduced sensation in the upper limbs distally, likely due to diabetic peripheral neuropathy. Initial treatment with a one-week course of antibiotics was started for a urinary tract infection-based culture and sensitivity and surgery was delayed till clearance of urinary tract infection to reduce the risk of implant infection. On the third day of admission, the patient developed acute urinary retention, necessitating Foley catheter insertion. He was later transferred to the rehabilitation ward and referred to neurology on day six for assessment due to the rapid progression of his peripheral neuropathy.

**Figure 1:**
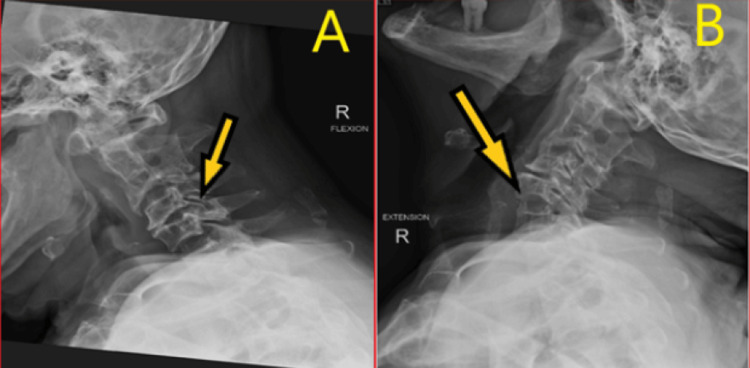
Pre-operative flexion and extension X-ray of cervical spine. A: Arrow points to mild retrolisthesis of C3 over C4. B: Arrow points to mild anterolisthesis of C4 over C5.

**Figure 2:**
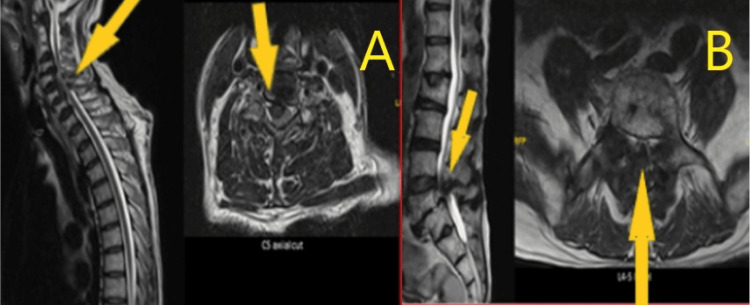
Pre-operative MRI whole spine. A: Multilevel cervical spondylotic changes, particularly severe at C4/C5 and C5/C6, with significant spinal canal stenosis, increased T2 cord signal, and cord narrowing suggesting possible myelomalacia as shown by arrows. B: Lumbar MRI showing multilevel spondylotic changes, with severe stenosis most pronounced at L3/L4 and L4/L5 as pointed by arrows.

Neurological examination showed reduced power in all four limbs (right side greater than left) and diminished sensation in both upper and lower limbs. Cervical spine X-rays with flexion and extension views revealed decreased vertebral height at multiple levels, mild retrolisthesis of C3 over C4, and anterolisthesis of C4 over C5 (Figure 1). MRI of the entire spine indicated multilevel cervical spondylotic changes, particularly severe at C4/C5 and C5/C6, with significant spinal canal stenosis, increased T2 cord signal, and cord narrowing suggesting possible myelomalacia and there were multilevel spondylotic changes in lumbar spine, with severe stenosis most pronounced at L3/L4 and L4/L5 (Figure 2). Upon review by the spine team, decreased power was noted in C8 myotomes bilaterally, along with reduced sensation in all four limbs (Table 1). Informed surgery consent was taken from the patient and was scheduled for C5 corpectomy and posterior decompression with fusion as an emergency procedure on the 21st post-fall day.

**Table 1:** Limb’s neurology examination findings on initial assessment, ASIA score D

Limb					
Right upper limb power and sensations	C5 5/5, +/++	C6 5/5, +/++	C7 5/5, +/++	C8 3/5, +/++	T1 2/5, +/++
Left upper limb power and sensations	C5 5/5, +/++	C6 5/5, +/++	C7 4/5, +/++	C8 3/5, +/++	T1 2/5, +/++
Right lower limb power and sensations	L2 5/5, +/++	L3 5/5, +/++	L4 5/5, +/++	L5 5/5, +/++	S1 5/5, +/++
Left Lower limb power and sensations	L2 5/5, +/++	L3 5/5, +/++	L4 5/5, +/++	L5 5/5, +/++	S1 5/5, +/++

On the 13th day of admission, the patient developed hematuria and was started on oral antibiotics for a urinary tract infection, delaying surgery until he fully recovered. A day prior to surgery, further deterioration was noted in upper limb strength (Table 2). On the 20th day, the patient underwent a staged procedure involving C5 corpectomy, insertion of an expandable titanium cage, anterior plating, C4/5 laminectomy, and fixation with C3 to C6 lateral mass screws and rods.

**Table 2:** Pre-operative upper limb power deterioration one day before surgery based on Medical Research Council (MRC) grading.

Limb					
Power right upper limb	C5 5/5	C6 3/5	C7 3/5	C8 3/5	T1 2/5
Power left upper limb	C5 5/5	C6 4/5	C7 4/5	C8 3/5	T1 2/5

**Figure 3:**
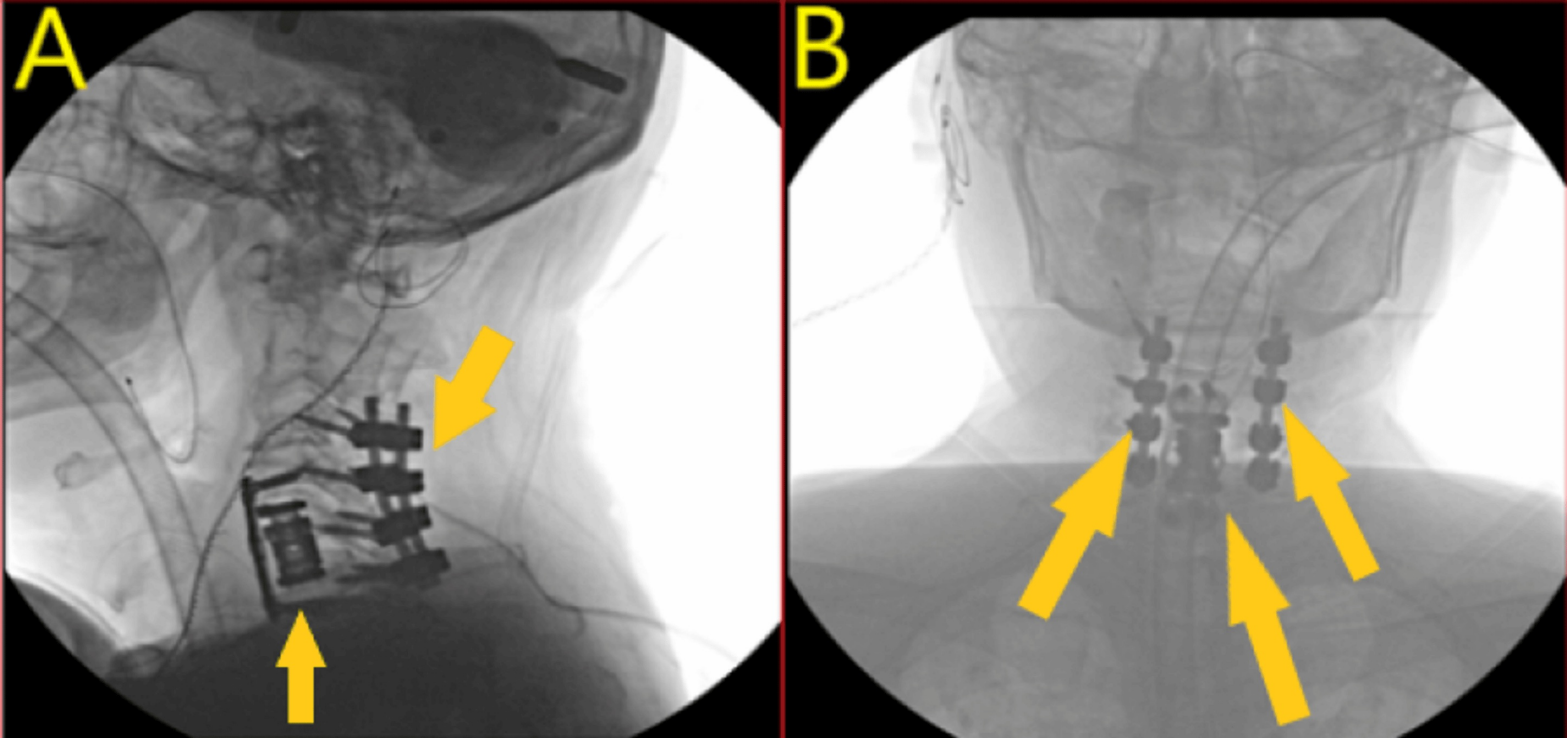
Post-operative X-ray cervical spine. A: X-ray cervical spine lateral views showing no malalignment of implants pointed to by arrows. B: X-ray cervical spine A/P views having no implant malalignment pointed to by arrows.

**Figure 4:**
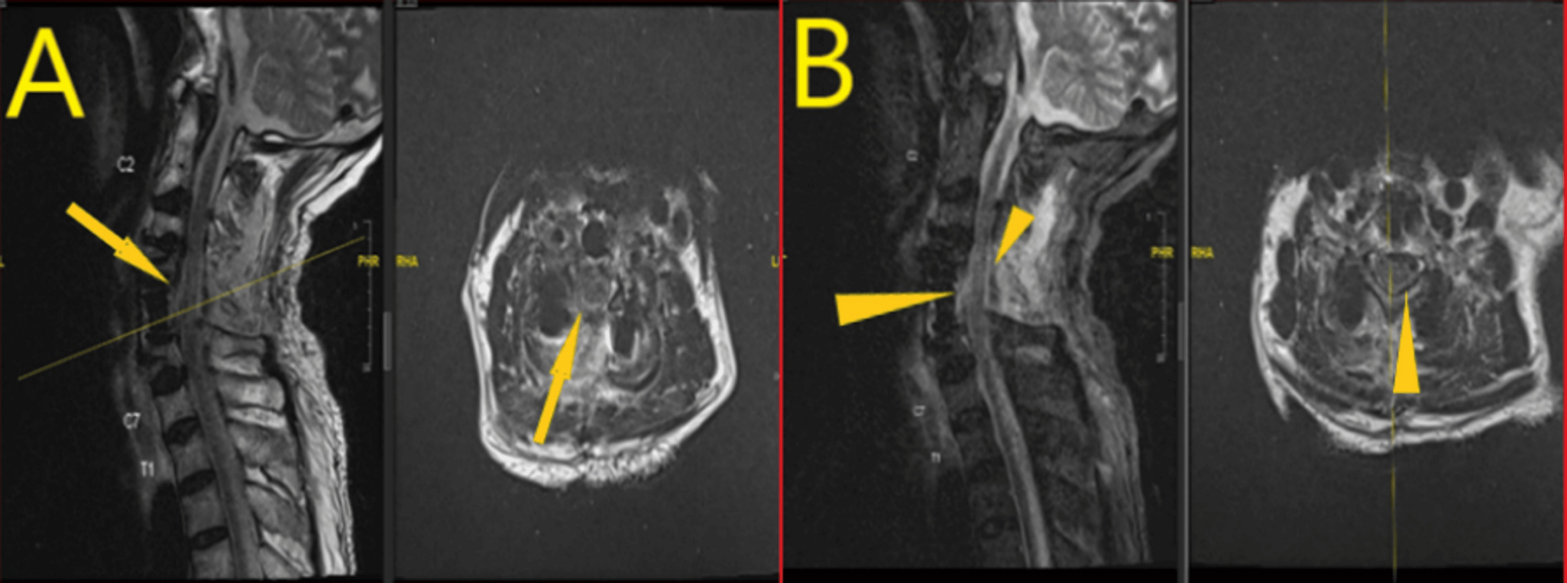
First post-operative MRI cervical spine after deterioration of right side power. A: T2-weighted MRI of the cervical spine revealing a rim of fluid collection at the surgical site with possible intradural extension and cord edema from C4-C6 as pointed to by arrows. B: MRI cervical spine TR independent multislice imaging showing more clear picture of rim of fluid at the surgical site shown by arrow heads.

No intraoperative complications occurred. Preoperative somatosensory evoked potential showed delayed bilateral latency and low amplitude in the lower limbs and left upper limb, while motor evoked potential responses were intact from C3-C6, with minimal responses in hands and feet. Postoperative somatosensory evoked potential and motor evoked potential findings remained consistent with the baseline. On the first postoperative day, neurological assessment showed improved upper limb strength (5/5 in all key myotomes, except 3/5 for bilateral T1, as shown in Table 3). X-ray of the cervical spine confirmed satisfactory implant positioning (Figure 3). However, on postoperative day 4, the patient experienced an episode of overnight confusion, which resolved by morning. The following day, delirium worsened, accompanied by new-onset right-sided weakness (Table 4). Urgent brain CT and cervical spine MRI were performed. The brain CT showed no acute intracranial hemorrhage or infarct, while MRI of the cervical spine revealed a fluid collection evident on T2 weighted image and no contrast enhancement at the surgical site with possible intradural extension and cord edema from C4-C6 (Figure 4). The patient was referred to general medicine for delirium management, attributed to multifactorial causes, including recent surgery, an unfamiliar hospital environment, and urinary tract infection. Antibiotics were escalated to IV meropenem.

**Table 3:** Post-operative improvement in upper limb power based on Medical Research Council (MRC) grading

Limb					
Power right upper limb	C5, 5/5	C6, 5/5	C7, 5/5	C8, 5/5	T1, 3/5
Power left upper limb	C5, 5/5	C6, 5/5	C7, 5/5	C8, 5/5	T1, 3/5

**Table 4:** Post-operative day four upper limb power deterioration based on Medical Research Council (MRC) grading

Limb					
Power right upper limbs myotome	C5, 2/5	C6, 3/5	C7, 3/5	C8, 3/5	T1, 3/5
Power right upper limbs myotome	C5, 5/5	C6, 5/5	C7, 5/5	C8, 5/5	T1, 5/5

**Figure 5:**
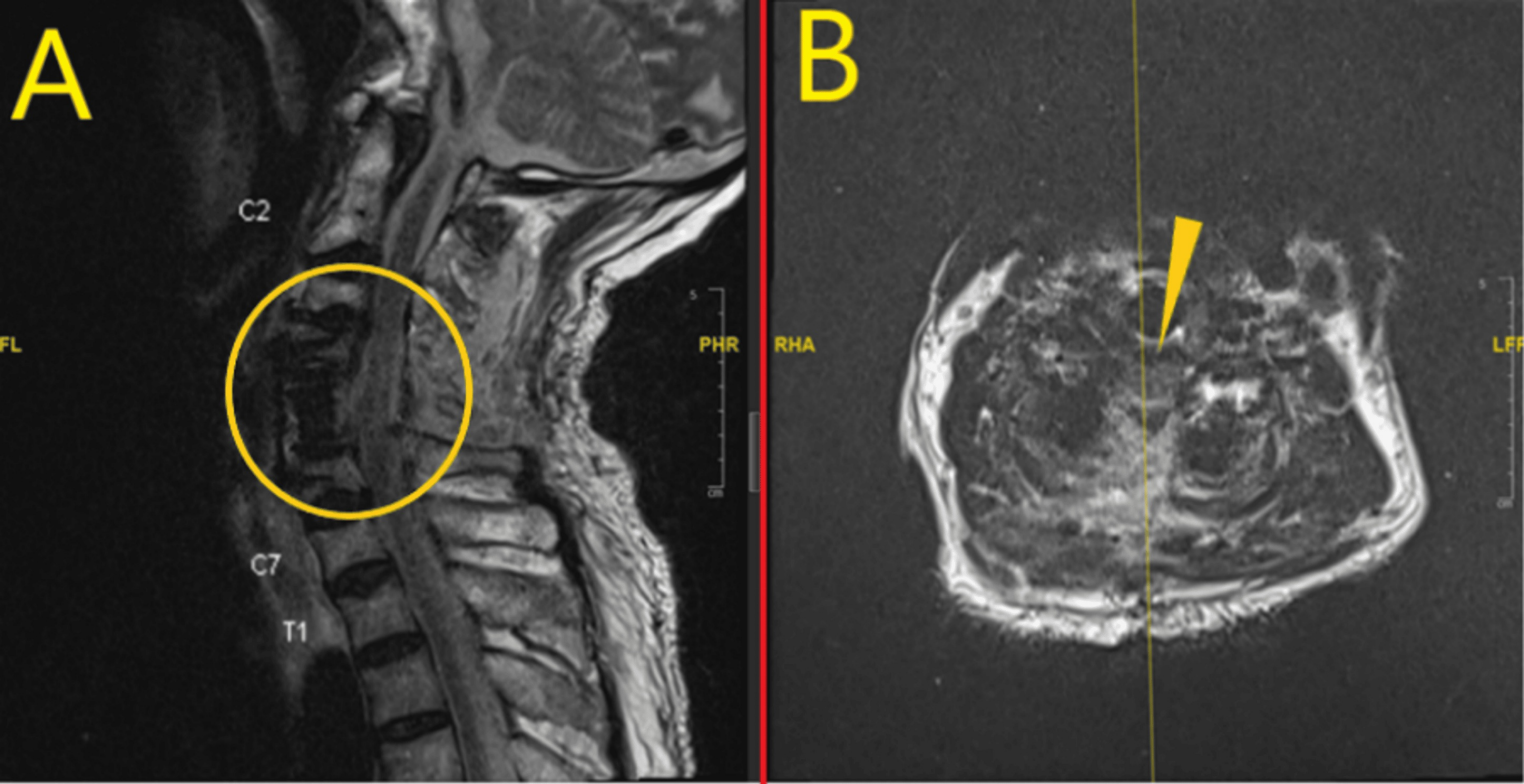
Repeat MRI cervical spine after bilateral foot drop. A: MRI cervical spine sagittal view; interval improvement in fluid collection at surgical bed around spinal cord and no spinal cord edema as shown in encircled area. B: MRI cervical spine axial T2 weighted image showing decreased fluids signals around the spinal cord and the cord is now more clearly visible as shown by arrowhead.

MRI/Magnetic Resonance Angiography (MRA) of the brain showed no acute infarction. IV dexamethasone was reintroduced on postoperative day nine for suspected cord reperfusion injury. By postoperative day 13, delirium had resolved, but neurological assessment revealed bilateral foot drop (1/5 power in bilateral L4 and L5 myotomes), although digital rectal examination was normal, with no signs of cauda equina syndrome. A repeat MRI of the cervical spine showed a slight reduction in the size of the fluid collection at the surgical site, with no evidence of intradural extension or abnormal cord edema (Figure 5). IV dexamethasone was tapered off on postoperative day 14, and a family conference was held to discuss the newly developed bilateral foot drop, likely due to aggravation of lumbar stenosis and listhesis symptoms after cervical spine decompression. The patient and family opted for surgery.

**Figure 6:**
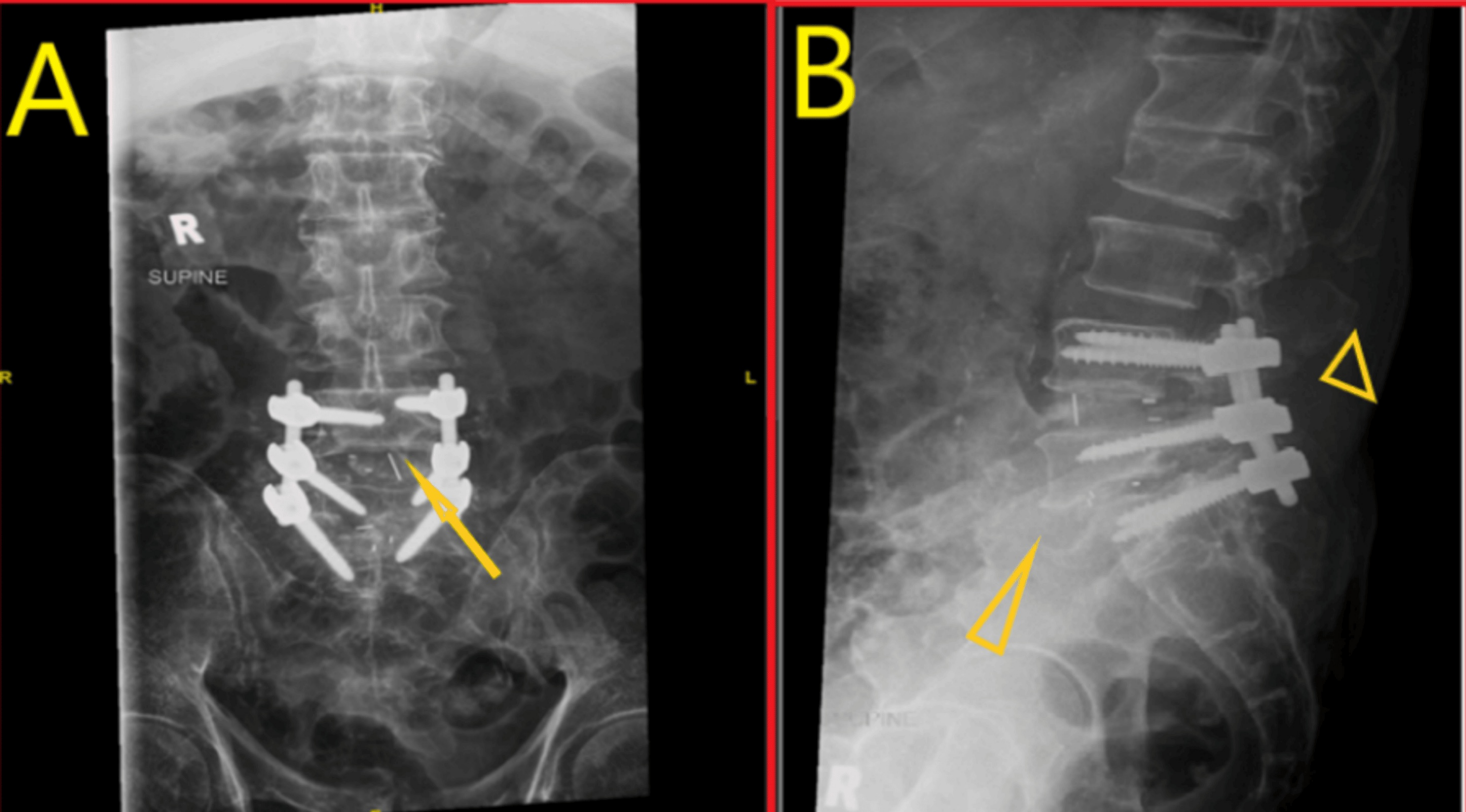
Post-operative X-ray lumbar spine. A: Posterior spinal instrumentation from L3-L5 levels with favorable location of interbody cage as pointed to by arrow. B: Grade 1-2 anterolisthesis at L4-5 level and aligned posterior implant as pointed by arrowheads.

For the second surgery informed consent was again taken from the patient. On postoperative day 16 of cervical spine surgery, the patient underwent L3/4 and L4/5 transforaminal lumbar interbody fusion (TLIF) with no intraoperative complications. Intraoperative somatosensory evoked potential and motor evoked potential remained consistent with baseline findings. Postoperatively, the patient was transferred to the general ward, but redeveloped delirium on the first postoperative day. Delirium workup was initiated, and treatment was provided per the general medicine team’s guidance. Over two weeks, the delirium resolved, and rehabilitation commenced. The patient was ultimately discharged to a nursing home per rehabilitation team recommendations. At discharge, the power was 2/5 in bilateral L4 and L5 myotomes (Table 5). Postoperative X-ray lumbar spine and the report showed posterior spinal instrumentation is noted from L3-L5 levels. There was grade 1-2 anterolisthesis at the L4-5 level, with slight retrolisthesis at L2-3. Likely chronic anterior wedging of the L1 body. Multilevel degenerative endplate osteophytes and facetal arthropathies are seen. Disc spaces appear reduced at T12-L1 and L4-5 levels as shown in Figure 6.

**Table 5:** Post lumbar 4/5 decompression and transforaminal lumbar interbody fusion (TLIF) surgery power of lower limbs at time of discharge based on Medical Research Council (MRC) grading

Limb					
Power right upper limb	C5, 4/5	C6, 5/5	C7, 5/5	C8, 4/5	T1, 5/5
Power left upper limb	C5, 5/5	C6, 5/5	C7, 5/5	C8, 5/5	T1, 5/5
Power right lower limb	L2, 5/5	L3, 5/5	L4, 2/5	L5, 2/5	S1, 4/5
Power left lower limb	L2, 5/5	L3, 5/5	L4, 2/5	L5, 2/5	S1, 4/5

## Discussion

This case primarily illustrates spinal tandem stenosis with superimposed WCS of the cervical spine having worsening power of the right upper limb and radiologically signal changes in the cervical spine after surgery, a rare but significant reperfusion injury. WCS, a condition involving acute reperfusion injury of chronically ischemic areas in the spinal cord, occurs after decompression procedures. Diagnosis hinges on excluding other causes. White cord syndrome or reperfusion injury of spinal cord is a rare clinical condition that occurs due to acute reperfusion injury of a chronically ischemic area of the spinal cord after either anterior or posterior decompression of the spinal cord. It is a diagnosis of exclusion. A patient having unexplained neurological deficit after decompressive surgery of spinal cord and having intramedullary hyper-intense area on MRI is diagnostic for white cord syndrome [20].

In the literature the incidence of radiological tandem spinal stenosis is documented as 8 to 60% while the symptomatic form ranges from 5-28%, respectively [21]. Regarding white cord syndrome, Chin et al. in 2013 described the first case of white cord syndrome and after this its incidence has increased as physicians are becoming more and more aware of it [22]. So JS et al. have described the features in table form of all cases of white cord syndrome from 2013 to 2023. All the cases were operated for cervical stenosis. The post-operative weakness is in the form of quadriplegia, hemiplegia, hemiparesis, anterior cord syndrome, monoplegia, and paraplegia. Out of 15 cases 11 patients showed full recovery or with some residual weakness while four patients did not recover [23].

The clinical picture is usually confusing in cases of tandem stenosis because the clinical findings in lower limbs are similar in the cases of tandem stenosis. Therefore, it is extremely important to recognize cervical and lumbar stenosis by carefully revising the history and physical examination findings for early detection and prevention of further late complications. In old age frequency of spinal stenosis increases because of more degenerative changes therefore more attention should be given to elderly patients having presentation of gait issues or neurogenic claudication. Early recognition by careful history and neurological examination and then direction-appropriate radiological investigations and if indicated early surgical management are the key elements of tandem spinal stenosis management. Those who do not fulfill the criteria of early surgical intentions should be followed regularly so that in case any intervention is needed in the future, it can be done in due time [18]. Because of the overlapping clinical picture of cervical myelopathy and lumbar spinal stenosis, the change in sensorimotor functions of lower limbs associated with positions like bicycling test, neurogenic claudication provoked by walking and prolonged standing can aid more accurately in diagnosis of concomitant lumbar spine stenosis [24-26].

It has been hypothesized that the key players in ischemic reperfusion injury of the spinal cord are oxidative and nitrosative stress mediating its effect through lipid peroxidation, protein degradation products, activation of inflammatory cascade activation and cell death [27]. Bagherzadeh et al. described important risk factors for white cord syndrome in their study which include cervical stenosis cases due to opacified posterior longitudinal ligament, prolonged operating hours and advanced age. Until now the treatment options which are mentioned in literature are keeping mean arterial pressure (MAP) > 85 in postoperative period, high dose IV methylprednisolone, and occasional usage of neurotropic agents to boost MAP and optimum rehabilitation. Seventy-five percent of patients can be able to walk with or without walking aids but 25% remain disabled. The most important outcome factor in whether the patient will achieve walking ability or not is the pre-operative Nurick grading [28].

Additionally, propofol also has a protective role by inhibiting nuclear factor kappa B (NF-kB) pathways leading to decreased expression of proinflammatory markers which leads to decreased inflammation thus improving the function of blood spinal cord barrier. Therefore, propofol should be the preferred anesthetic agent instead of volatile gases [29]. A study by Vidal et al. done on a mouse model described that the risk of reperfusion injury increases proportionally to the delay in decompressive surgery. The more the surgery is delayed the more serious will be consequences of reperfusion injury. One of the mechanisms is that increased blood flow occurs after delayed decompression of chronically ischemic areas [30,31].

Kumar V et al. described in their case report quadriplegia with decreased sensations in the downstream dermatomes associated with white cord syndrome. On one-year post-operative follow-up after intense physiotherapy the patient was able to walk with support along with residual upper limb paresthesia. Most of the studies have shown that initially the partial improvement occurs then followed by a period of steady state until the final improvement occurs. However one-fourth of the patients do not show any further improvement [32].

## Conclusions

Although tandem stenosis cases are often asymptomatic, they warrant close consideration, especially in elderly patients. Thorough initial history and examination are critical for prompt diagnosis and timely radiological assessment. For patients with signs of ischemic cervical myelopathy, the risk of reperfusion injury must be communicated preoperatively, highlighting potential postoperative neurological deficits. Intravenous methylprednisolone, optimized MAP, and structured rehabilitation remain the cornerstones of WCS treatment. The majority of WCS patients can expect significant recovery, although a few of them may face lasting impairment. Also, close attention and high index of clinical suspicion should be paid to tandem stenosis in other areas of the spine and patients should be warned about the potential for future surgeries for tandem stenosis or if facilities allow, to be addressed simultaneously or in a staged manner.
